# Subglottic Stenosis After Radioactive Iodine Treatment for Graves' Disease: A Case Report

**DOI:** 10.1002/hed.70157

**Published:** 2025-12-31

**Authors:** Claire Ettlin, Roland Giger, Yves Jaquet, Samuel Tschopp

**Affiliations:** ^1^ Department of Otorhinolaryngology, Head and Neck Surgery Inselspital, University Hospital and University of Bern Bern Switzerland; ^2^ Service d'ORL et chirurgie cervico‐faciale, Département de chirurgie Réseau hospitalier neuchâtelois Neuchâtel Switzerland

**Keywords:** airway, complication, radioactive iodine treatment, subglottic stenosis

## Abstract

**Background:**

Subglottic stenosis (SGS) is a narrowing of the airway below the glottis and may be congenital or acquired. While prolonged intubation is the most common cause of acquired SGS, other etiologies remain incompletely understood.

**Methods:**

A 37‐year‐old woman presented with a four‐year history of mild progressive stridor, dysphonia, and chronic cough. Her medical history was notable for Graves' disease, treated initially with long‐term carbimazole followed by radioactive iodine (RAI). She had never been intubated and had no autoimmune or rheumatologic disease. Awake fiberoptic endoscopy raised suspicion of SGS, which was confirmed by preoperative magnetic resonance imaging.

**Results:**

Suspension microlaryngoscopy with biopsy and dilation revealed a Myer‐Cotton grade II SGS with an irregular aspect and exposed cartilage. Histopathology demonstrated chronic inflammation, fibrosis, and reactive epithelial changes consistent with radiation‐induced injury. After a second endoscopic dilation, only a minor anterior synechia remained, without evidence of recurrent stenosis.

**Conclusions:**

This case report highlights a temporal association between RAI therapy and the subsequent development of SGS, supported by histopathological findings suggestive of radiation‐induced tissue injury. In the absence of other established risk factors, RAI treatment may represent a rare but clinically relevant cause of acquired SGS, a complication not previously reported in the literature.

## 1 Introduction

Subglottic stenosis (SGS) is a rare condition that may be either congenital or acquired. In adults, acquired SGS most commonly results from prolonged endotracheal intubation or autoimmune disease. In a substantial proportion of cases, however, no clear etiology can be identified, and these are classified as idiopathic SGS. Idiopathic SGS has an estimated incidence of 1 in 400,000, and it predominantly affects women between the third and fifth decades of life [1].

Clinical presentation depends on the degree of airway narrowing and may include progressive dyspnea, stridor, chronic cough, and voice changes. Diagnosis work‐up typically involves laryngotracheoscopy with biopsy, cross‐sectional imaging, and laboratory testing. Management strategies depend on the severity and extent of the stenosis and range from endoscopic interventions, such as bougie or balloon dilatation with or without CO_2_ laser incisions, to open reconstructive surgery in severe cases [1, 2, 3].

We report the case of a woman who developed SGS following radioactive iodine (RAI) therapy for Graves' disease, in the absence of other established risk factors.

## 2 Case Report

A 37‐year‐old woman presented with a four‐year history of progressive stridor, shortness of breath, chronic cough, and dysphonia. She had no history of endotracheal intubation, airway trauma, autoimmune or rheumatological disease. Her medical history was notable for Graves' disease, initially treated with long‐term carbimazole, followed by a single dose of RAI therapy (442.5MBq I‐131) 3 years before symptom onset. She had experienced four pregnancies complicated by hyperemesis gravidarum.

**FIGURE 1:**
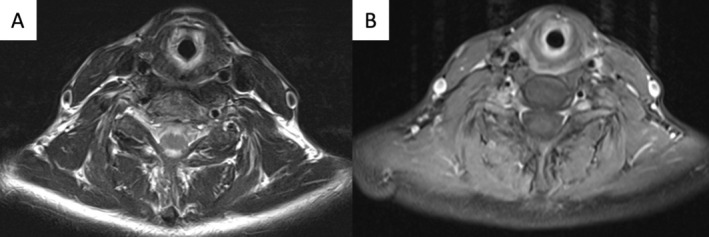
Magnetic resonance imaging of the subglottic region: T2‐weighted image (Panel A) shows a subglottic circumferential stenosis with mucosal thickening and T1‐weighted image (Panel B) demonstrates diffuse contrast enhancement.

Transnasal awake fiberoptic endoscopy revealed a subglottic narrowing suspicious of SGS. Magnetic resonance imaging demonstrated a circumferential subglottic narrowing with mucosal thickening and diffuse contrast enhancement (Figure 1). Serological tests for autoimmune disease were negative.

**FIGURE 2:**
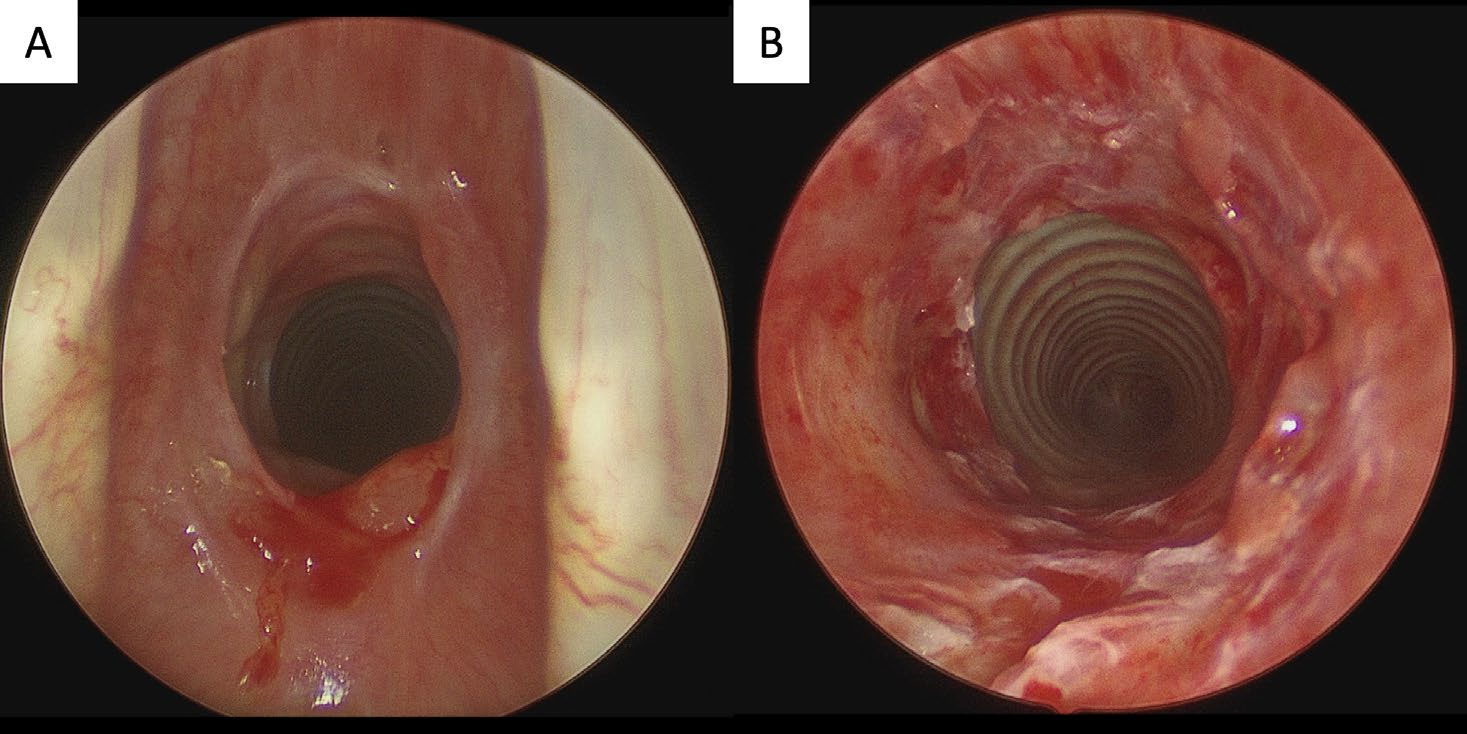
First suspension microlaryngoscopy: Panel A shows the subglottic stenosis after biopsy. Panel B demonstrates the subglottis after CO_2_‐laser incision and subglottic bougie dilatation from Charrière 29–39 (9.7–13 mm).

**FIGURE 3:**
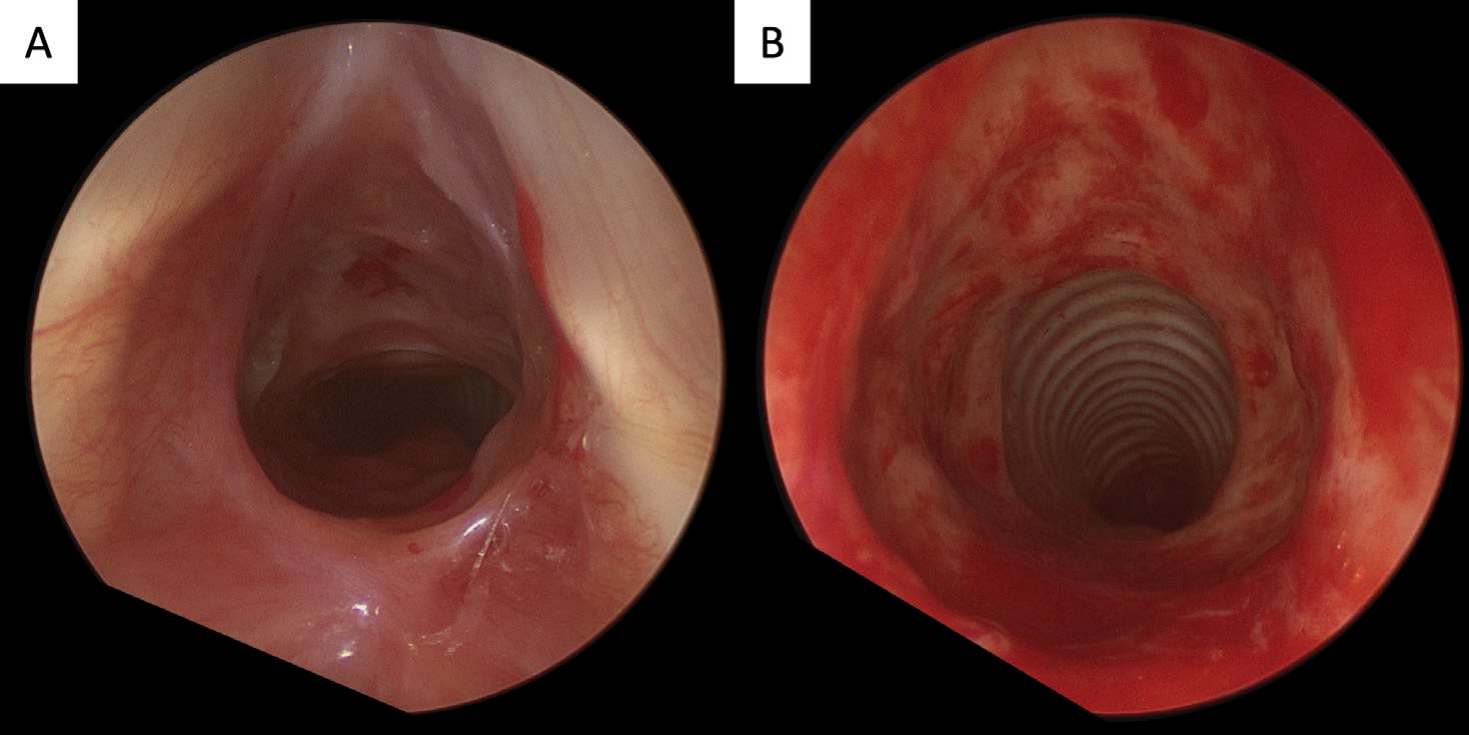
Second suspension microlaryngoscopy: Panel A shows the stenosis before intervention and panel B after subglottic bougie dilatation from Charrière 32–41 (10.7–13.7 mm).

The patient underwent suspension microlaryngoscopy with biopsy and bougie dilatation from Charrière 29–39 (9.7–13 mm). The stenosis was classified as Myer‐Cotton grade II, extending 14 mm in length and beginning 5 mm below the vocal cords (Figure 2). Exposed cartilage was observed within the stenotic segment. Histopathological analysis of the biopsy revealed chronic granulomatous inflammation with ulceration, fibrosis, and reactive epithelial atypia, findings suggestive of radiation‐induced changes. Postoperatively, the patient received proton pump inhibitors, antitussives, and saline inhalation therapy. A second endoscopic treatment with bougienage was performed 6 weeks later, increasing the stenosis diameter from Charrière 32–41 (10.7–13.7 mm, Figure 3).

Follow‐up endoscopy at 3 months revealed no restenosis but a small residual anterior glottic synechia, which was managed conservatively. At the 6‐month follow‐up after dilatation, the patient reported minor residual voice changes and cough attributable to the residual synechia and remained otherwise asymptomatic, with normal respiration and no exercise‐induced dyspnea.

## 3 Discussion

Subglottic stenosis is rare and most frequently caused by intubation‐related injury or autoimmune disease. Nevertheless, a considerable proportion of cases remain idiopathic. In the present case, the patient had no history of intubation, trauma, autoimmune disease, or gastroesophageal reflux, all recognized contributors to SGS [1]. Although pregnancy and vomiting have been suggested as possible modifiers of disease course, they are not established primary etiologies of SGS, but may contribute to recurrence [4, 5].

The gradual onset of symptoms followed RAI therapy for Graves' disease, raising suspicion for a causal relationship. Magnetic resonance imaging demonstrated stenosis at the level of the thyroid gland, and histopathological findings were consistent with radiation‐induced tissue changes, supporting a potential pathophysiological link between prior RAI therapy and the development of SGS.

RAI therapy is an established and generally well‐tolerated treatment for refractory Graves' disease. While radiation‐induced airway complications have been described, including chondronecrosis and stenosis after brachytherapy with RAI for a mediastinal tumor in the distal trachea, as well as after external beam irradiation in the proximal trachea, they remain exceedingly rare [6, 7, 8, 9]. To our knowledge, this is the first reported case of SGS after RAI treatment.

Our patient responded well to two successful endoscopic bougie dilatations, achieving sustained airway patency and symptom relief. Although recurrence remains a concern, endoscopic techniques are generally preferred in mild to moderate SGS due to their minimally invasive nature and shorter recovery times [10].

This case highlights RAI therapy as a potential, under‐recognized cause of SGS. Given the latency of onset and nonspecific presentation of early symptoms, clinicians should maintain a high index of suspicion for SGS in patients with unexplained upper airway symptoms and a history of RAI treatment, particularly when more common etiologies have been excluded.

## 4 Conclusion

We report a rare case of SGS developing after RAI therapy for Graves' disease in a patient without established risk factors such as intubation, autoimmune disease, or gastroesophageal reflux. The chronological association, radiological evidence, and histopathological findings support radiation‐induced injury as a plausible mechanism. Although this association has not previously been widely recognized, RAI should be considered among the potential causes of acquired SGS.

## Funding

The authors have nothing to report.

## Consent

Written informed consent was obtained from the patient for publication of this case report and accompanying images. All reasonable efforts were made to ensure patient anonymity.

## Conflicts of Interest

The authors declare no conflicts of interest.

## Data Availability

The data that support the findings of this study are available from the corresponding author upon reasonable request.

## References

[1] C. Aravena , F. A. Almeida , S. Mukhopadhyay , et al., “Idiopathic Subglottic Stenosis: A Review,” Journal of Thoracic Disease 12 (2020): 1100–1111, 10.21037/jtd.2019.11.43. 32274178 PMC7139051

[2] R. T. Cotton , “Management of Subglottic Stenosis,” Otolaryngologic Clinics of North America 33 (2000): 111–130, 10.1016/s0030-6665(05)70210-3. 10637347

[3] H. C. Grillo , E. J. Mark , D. J. Mathisen , and J. C. Wain , “Idiopathic Laryngotracheal Stenosis and Its Management,” Annals of Thoracic Surgery 56 (1993): 80–87, 10.1016/0003-4975(93)90406-8. 8328880

[4] L. Dornan , M. Crawford , P. Milligan , and P. A. Ward , “Subglottic Stenosis in Pregnancy: A Case Report,” Journal of Oral and Maxillofacial Anesthesia 4 (2025): 9, 10.21037/joma-25-4.

[5] A. S. Awadallah , F. W. Fearington , Y. H. Khalil , et al., “Pregnancy and Parity as Risk Factors for Recurrence in Idiopathic Subglottic Stenosis,” Otolaryngology‐Head and Neck Surgery 173 (2025): 447–452, 10.1002/ohn.1255. 40211691

[6] A. H. Alraiyes , M. C. Alraies , and A. Abbas , “Radiation‐Associated Airway Necrosis,” Ochsner Journal 13 (2013): 273–275. 23789018 PMC3684341

[7] Y. Takiguchi , H.‐O. Okamura , K. Kitamura , and S. Kishimoto , “Late Laryngo‐Tracheal Cartilage Necrosis With External Fistula 44 Years After Radiotherapy,” Journal of Laryngology and Otology 117 (2003): 658–659, 10.1258/002221503768200048. 12956925

[8] H. Suzuki , T. Tanifuji , I. Hasegawa , and T. Fukunaga , “A Forensic Autopsy Case of Death From Laryngeal Stenosis due to a Late Complication of Radiotherapy,” Legal Medicine 20 (2016): 15–17, 10.1016/j.legalmed.2016.03.004. 27161915

[9] N. Queenan , J. Trivedi , D. Bertoni , S. H. Siddiqui , A. Yap , and K. M. Tibbetts , “Characterizing Radiation‐Related Laryngotracheal Stenosis,” American Journal of Otolaryngology 46 (2025): 104643, 10.1016/j.amjoto.2025.104643. 40311495

[10] E. Lavrysen , G. Hens , P. Delaere , and J. Meulemans , “Endoscopic Treatment of Idiopathic Subglottic Stenosis: A Systematic Review,” Frontiers in Surgery 6 (2020): 75, 10.3389/fsurg.2019.00075. 31998744 PMC6965146

